# Advances in the Gut‐Lung Axis and Bronchial Asthma: From Mechanisms to Therapeutic Potential

**DOI:** 10.1002/clt2.70128

**Published:** 2025-11-27

**Authors:** Ting Zheng, Yi Huang, Hongmei Yao

**Affiliations:** ^1^ Respiratory Medicine Zunyi Medical University Zunyi Guizhou China; ^2^ Respiratory Medicine Guizhou Provincial People's Hospital Guiyang City Guizhou Province China

**Keywords:** bronchial asthma, gut‐lung axis, gut microbiota, immunity

## Abstract

**Background:**

Bronchial asthma (hereafter referred to as asthma) is a heterogeneous disease characterized by chronic airway inflammation, airway hyperresponsiveness (AHR), and reversible airflow limitation. Recent advancements in the “gut‐lung” axis concept have elucidated the intricate immunometabolic crosstalk between the gastrointestinal tract and pulmonary system, offering novel insights into the pathogenesis and therapeutic strategies for asthma.

**Objective:**

Exploring the research progress of the “gut‐lung” axis in bronchial asthma, providing new insights into the pathogenesis and treatment of asthma.

**Methods:**

This article reviewed a large number of relevant studies, elucidating the role of the “gut‐lung” axis in bronchial asthma, and further discussing the potential of the microbiota in the treatment of bronchial asthma.

**Conclusion:**

Gut microbiota and their metabolites exert profound effects on pulmonary immune homeostasis through immune modulation, metabolic signaling, and neuroendocrine pathways, which are critically implicated in asthma pathogenesis. Conversely, systemic immune dysregulation in asthma may reciprocally induce intestinal barrier dysfunction. Asthma is a variable disease here. It may be that some of this variability relates to different diets or environmental/gut exposures although this needs to be confirmed in human studies. This review comprehensively delineates the mechanistic role of the gut‐lung axis in asthma, encompassing microbiota‐immune system interactions, regulatory effects of microbial‐derived metabolites such as short‐chain fatty acids (SCFAs) and bile acids, and microbiota‐targeted therapeutic approaches including probiotics, dietary interventions, and metabolite‐based therapies. Furthermore, we also discuss the limitations and future development directions of current research to provide new ideas for precision treatment of asthma.

## 1 Introduction

Asthma is a common chronic respiratory disease characterized by marked heterogeneity and variability. This variability may be associated with distinct dietary patterns or environmental/gut microbial exposures, although the causal relationships remain to be clearly established in human studies. Its pathogenesis involves a complex interplay of genetic, environmental, and immune factors. According to estimates by the World Health Organization (WHO) and the Global Initiative for Asthma (GINA), approximately 300 million individuals currently suffer from asthma globally, with projections indicating a rise to 400 million by 2025 [1]. Asthma‐related mortality accounts for an estimated 250,000 deaths annually [2]. Pathophysiologically, asthma‐related inflammatory responses can be classified into two major categories: type 2 (T2) and non‐type 2 (non‐T2) inflammation. T2 inflammation is further subdivided into allergic and non‐allergic endotypes, driven by eosinophil infiltration, Th2 cell activation, proliferation of group 2 innate lymphoid cells (ILC2s), and IL‐4/IL‐5/IL‐13 cytokine cascades. In contrast, non‐T2 inflammation exhibits heterogeneous mechanisms involving Th1/Th17 lymphocyte activation pathways and alternative immune cascades [3, 4]. The conventional management of asthma primarily involves glucocorticoids and β2‐adrenoceptor agonists, yet a subset of patients continues to exhibit refractory symptoms. Studies indicate that while the majority of pediatric asthma patients achieve well‐controlled symptoms through low‐dose inhaled corticosteroids (ICS) or self‐management interventions, approximately 10% of cases persist as refractory asthma, demonstrating glucocorticoid resistance to corticosteroid therapy [5, 6]. Therefore, further elucidation of the pathogenesis of refractory asthma is required to provide novel therapeutic approaches for asthma management.

Emerging research on the gut microbiota has revealed its central role in immune modulation. Transmitted maternally through breastfeeding, the microbiota serves as a pivotal regulator of the gut‐lung axis functionality [7]. Emerging paradigms of the gut‐lung crosstalk provide novel perspectives on asthma mechanisms. The gut microbiota establishes a bidirectional regulatory network with the pulmonary system through microbial metabolites, immune cell trafficking, and signaling molecules (Such as cytokines, neurotransmitters, etc.) [8]. This interaction plays an important role in the occurrence and development of asthma, and is expected to provide new opportunities for the diagnosis and treatment of asthma. This review elucidates mechanistic contributions of gut microbiota and the gut‐lung axis to bronchial asthma pathogenesis, while exploring microbiota‐targeted interventions as potential therapeutic modalities. It should be noted that most current insights into the gut‐lung axis mechanisms in asthma come from well‐established animal models. Although these models are indispensable for exploring causal relationships and molecular pathways, this article will clearly specify the research background throughout and will particularly emphasize data from human clinical studies.

## 2 Immunological Mechanisms Underlying Asthma

**FIGURE 1:**
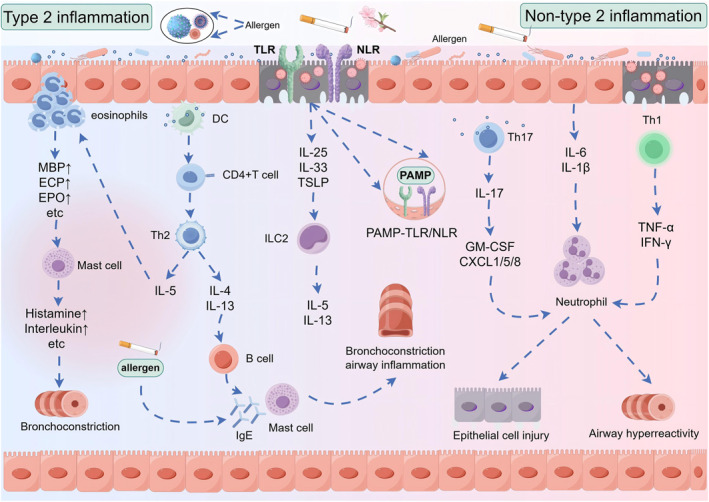
Immune mechanisms of asthma. Type 2 inflammatory asthma: When allergens stimulate the airways, DCs present the allergens to naïve T cells and promote their differentiation into Th2 cells, which further secrete IL‐5, IL‐4, and IL‐13. IL‐4 and IL‐13 activate B cells to produce IgE, which binds to mast cells. Upon re‐exposure to the same allergen, it acts on mast cells via IgE, inducing bronchoconstriction and airway inflammation. IL‐5 promotes eosinophil recruitment to the lungs, releasing mediators such as MBP, which stimulates mast cells to release factors like histamine and interleukins, causing bronchoconstriction. Additionally, PAMP in allergens form PAMP‐TLR/NLRpatterns with TLRs and NLRs in airway epithelium, inducing epithelial cells to produce cytokines like TSLP, IL‐25, and IL‐33. These activate ILC2 to produce Th2 cytokines such as IL‐5 and IL‐13, inducing Th2‐mediated pulmonary inflammation. Non‐Type 2 inflammatory asthma mechanism: Under allergen stimulation, airway epithelium releases pro‐inflammatory cytokines like IL‐6 and IL‐1β. Concurrently, Th17 cells produce IL‐17, which stimulates epithelial cells and fibroblasts to release neutrophil chemokines CXCL1/5/8 and GM‐CSF, recruiting neutrophils to the lungs and inducing epithelial cell damage. Furthermore, infection and epithelial damage promote Th1 cell maturation and secretion of Th1 cytokines, such as TNF‐αand IFN‐γ. These can synergize with IL‐17 cytokines to promote neutrophil recruitment.

Asthma is an airway inflammatory disease characterized by distinct phenotypes. Based on Th2 inflammation status, it can be classified into Th2‐high and Th2‐low inflammatory phenotype asthma. Th2‐high asthma is characterized by eosinophilic airway inflammation, also known as type 2 inflammation, primarily mediated by Th2 and ILC2 cells and involving pathways such as IL‐4/IL‐13, IL‐5, and IL‐25/IL‐33/TSLP. Th2‐low asthma includes neutrophilic asthma and paucigranulocytic asthma, also termed non‐type 2 inflammation, primarily mediated by neutrophils and involving pathways such as TH1, IFN‐γ/TNF‐α, and TH17 [9]. Figure 1 summarizes the immune mechanisms of asthma.

The mechanism of Type 2 inflammatory asthma: Upon allergen exposure in the airways, dendritic cells (DCs) present processed allergens to Th2 lymphocytes, which subsequently secrete interleukin‐5 (IL‐5), IL‐4, and IL‐13. IL‐4 and IL‐13 activate B lymphocytes, driving their differentiation into IgE‐producing plasma cells. Synthesized IgE binds to high‐affinity receptors (FcεRI) on mast cells, completing sensitization. Upon re‐exposure to the same allergen, cross‐linking of IgE‐FcεRI complexes triggers mast cell degranulation, releasing mediators including leukotrienes, histamine, endothelin, prostaglandins, and thromboxane A2. These mediators induce bronchoconstriction and perpetuate airway inflammation. Concurrently, IL‐5 mediates eosinophilopoiesis and recruits eosinophils to pulmonary tissues. Activated eosinophils release cytotoxic granule proteins, notably major basic protein (MBP), which further stimulates mast cell histamine release and interleukin production, exacerbating bronchoconstrictive responses. Additionally, upon damage or infection, airway epithelial cells secrete alarmins, such as the cytokines thymic stromal lymphopoietin (TSLP), IL‐25, and IL‐33. These cytokines activate type 2 innate lymphoid cells (ILC2s), which produce Th2‐associated cytokines (IL‐5 and IL‐13) independent of adaptive immunity. This ILC2‐Th2 axis synergistically amplifies type 2 pulmonary inflammation through eosinophil recruitment, mucus hypersecretion, and airway remodeling [10, 11].

The mechanism of non‐type 2 inflammatory asthma: Upon allergen stimulation, the airway epithelium releases proinflammatory cytokines such as IL‐6 and IL‐1β. Concurrently, Th17 cells produce IL‐17, which stimulates epithelial cells and fibroblasts to secrete neutrophil chemoattractants CXCL1/5/8 and granulocyte‐macrophage colony‐stimulating factor (GM‐CSF). These mediators recruit neutrophils to the lungs, inducing epithelial cell injury. Additionally, infections and epithelial damage promote Th1 cell maturation and secretion of Th1 cytokines, including tumor necrosis factor‐alpha (TNF‐α) and interferon‐gamma (IFN‐γ). These cytokines synergize with IL‐17 to further enhance neutrophil recruitment and upregulate Ca^2+^ signaling in airway smooth muscle, thereby inducing airway hyperresponsiveness [12].

## 3 Crosstalk Between Lung and Gut Microbiomes

Although the lungs and intestines are two distinct organs, they are closely interconnected through a complex bidirectional network known as the “gut‐lung” axis. The core of this axis lies in their shared microbiota. As the largest and most complex ecosystem of microorganisms in the human body, the gut microbiome continuously influences distal pulmonary immunity and the inflammatory milieu via the production of metabolites, modulation of immune cells, and release of inflammatory mediators [13]. A healthy and balanced gut microbiota helps suppress excessive pulmonary inflammation and enhances resistance to pathogens. Conversely, gut dysbiosis may exacerbate pulmonary diseases—such as asthma, chronic obstructive pulmonary disease, and even respiratory infections—through immune mechanisms. Similarly, pulmonary inflammation and infection can reciprocally affect the gut via systemic effects, altering the composition and function of its microbiota [14].

Severe asthma is an asthma phenotype that responds poorly to standard therapy with inhaled corticosteroids and long‐acting β2‐agonists. It is characterized by suboptimal symptom control, frequent exacerbations, increased exposure to systemic corticosteroids, and a poor prognosis [15]. Research indicates that the colonization of the gut microbiome is crucial for the normal development of the immune system, and disruption of this process is closely linked to the onset and progression of asthma [16]. In patients with severe asthma, the epithelial gene set signature associated with Th17 cells—a hallmark of non‐type 2 inflammation—correlates with an abnormally elevated abundance of *Proteobacteria* [17]. This dysbiosis can further worsen asthma control and exacerbate neutrophilic airway inflammation [18, 19].

Alterations in the gut microbiota of severe asthma patients primarily include reduced alpha diversity, decreased relative abundance of beneficial microorganisms, and a reduction or even depletion of short‐chain fatty acid‐producing bacteria [20]. A study by Aslam et al. showed that higher alpha diversity of bacteria such as *Bifidobacterium*, *Faecalibacterium*, and *Roseburia* has a protective effect against asthma. In contrast, a lower relative abundance of *Bacteroides* and certain fungi, including *Malassezia*, has been associated with asthma [21]. Epithelial barrier dysfunction is both a characteristic and a pathological mechanism of airway inflammation in asthma patients. Compared to non‐asthmatic individuals, asthmatic patients exhibit impaired bronchial epithelial barrier function, which facilitates the entry of pathogens or allergens into airway tissues, activates immune responses, and leads to asthma exacerbations [22]. Studies have shown that short‐chain fatty acids can prevent and restore impaired airway epithelial barrier function. Research by Richards et al. demonstrated that short‐chain fatty acids enhance the barrier function of bronchial epithelial cells by increasing the expression of tight junction proteins and can restore barrier function impaired by stimulation with IL‐4, IL‐13, and house dust mite extract [23]. Furthermore, the carriage of antibiotic resistance genes in the gut microbiota is associated with asthma risk and exacerbation. In infants, microbial signatures linked to asthma development are also associated with increased richness of antibiotic resistance genes in the gut microbiome, and these differences in antibiotic resistance gene carriage are primarily driven by *Escherichia coli* [24]. Therefore, gut microbiota dysbiosis may exacerbate the severity of severe asthma by altering the host's immune status or systemic metabolic environment.

## 4 Mechanistic Studies of the Gut‐Lung Axis in Asthma

### 4.1 Composition of the Microbiota of the Lungs and Intestines

Microbial colonization commences at birth, marking a dynamic adaptation phase of infant metabolic programming and immune system ontogeny [25]. A critical developmental window exists during early life stages, during which microbiota perturbations may predispose to later disease trajectories [26]. Normobiotic intestinal microbiota exert multifaceted beneficial functions, including immune response potentiation and maintenance of intestinal microenvironmental homeostasis. Conversely, dysbiosis contributes to disease pathogenesis through metabolic and/or immune‐mediated pathways, potentially arising from depletion of beneficial microbes, pathobiont overexpansion, or loss of microbial diversity, which may disrupt mucosal immune ontogeny [27]. Emerging evidence indicates that the impact of gut microbiota and their metabolites on intestinal mucosal immunity influences immune responses at distal mucosal sites (such as pulmonary) [28, 29]. Therefore, advancing our understanding of the compositional and functional interplay between intestinal and pulmonary microbial communities is essential for deciphering the gut‐lung axis circuitry.

The composition of the microbiota is influenced by multiple factors early in life, such as the type of delivery, date of birth, mode of birth, feeding method and time of weaning, as well as age, lifestyle, eating habits, etc [30]. It is estimated that there are more than 100 trillion symbiotic microbial clusters inhabiting the human gastrointestinal tract, the vast majority of which are bacteria, mainly *Firmicutes* (such as *Lactobacillus*, *Bacillus* and *Clostridium*), *Bacteroides* (such as *Bacteroides*), in addition to *Proteobacteria* (such as *Escherichia*) and *Actinomycetes* (such as *Bifidobacteria*) [31]; But there are also fungi, viruses, including multiple organisms such as bacteriophages, archaea, protists, and worms. The colon, as the most densely colonized part, has an estimated density of 10^11^ to 10^12^ bacteria per milliliter [32, 33]. Previously, it was thought that the lungs were sterile, but further discoveries have led to the existence of microbial communities in the lungs [34]; Compared to the gut microbiota, the ecological scale of the lung microbiota has shrunk significantly, containing 10^3^ to 10^5^ bacteria per milliliter [35]. Due to the long‐term exposure of the lungs to the external environment and their unique topology, the lung microbiota is in a state of dynamic change [36]. The lungs of healthy people mainly contain six phyla: *Firmicutes*, *Proteobacteria*, *Bacteroides*, *Euryarchaeota*, *Acidobacterium* and *Actinomycetes* [37], among which *Firmicutes* and *Bacteroidetes* are the main groups [38], while the diversity of *Proteobacteria* increases in asthma patients [39].

In a Canadian longitudinal birth cohort study, Arrieta et al. analyzed fecal samples from 319 infants at 3 and 12 months of age, demonstrating transient gut microbial dysbiosis during the first 100 postnatal days in infants at‐risk for asthma. These high‐risk infants exhibited significant depletion of bacterial genera *Lachnospira*, *Veillonella*, *Faecalibacterium*, and *Roseburia*. Notably, supplementation of *Veillonella* spp. in germ‐free murine models markedly reduced levels of pro‐inflammatory cytokines associated with severe asthma (IL‐17A, IL‐6, TNF‐α) [40]. A Turkish cross‐sectional study further corroborated these findings, revealing lower counts of *Akkermansia muciniphila* and *Faecalibacterium prausnitzii* in 92 asthmatic children aged 3–8 years compared to healthy controls [41]. Evidence indicates that human milk promotes the proliferation of *Bifidobacterium* and *Lactobacillus* spp, thereby modifying the metabolic potential of the gut microbiota and ultimately modulating immune system development to confer allergy prevention [42, 43]. A Canadian prospective cohort study demonstrated that direct breastfeeding, compared to formula feeding or indirect breast milk exposure, confers protection against asthma risk [44]. Furthermore, studies reveal that elevated relative abundance of *Veillonella* spp. coupled with reduced levels of *Roseburia*, *Blautia*, and *Flavonifractor* at 1 year of age has been associated with an increased risk of asthma diagnosis by 5 years of age [45]. The study by Kei et al. revealed a dose‐dependent association between escalating pediatric asthma risk and elevated relative abundance of specific fungal taxa, including *Candida*, *Rhodotorula*, *Debaryomyces*, *Meyerozyma*, *Nigrospora*, and *Saccharomyces*, concomitant with reduced *Malassezia* colonization [46]. In summary, different microbial genera of intestinal flora can affect the occurrence and development of bronchial asthma.

### 4.2 Gut Microbiota and Immune Regulation

#### 4.2.1 Imbalance in Microbiota Diversity

The gut microbiota constitutes a diverse and intricate microecosystem that serves as a key modulator of the “gut‐lung” axis and plays a pivotal role in maintaining host homeostasis [47, 48]. Under homeostatic equilibrium, a normobiotic gut microbiota prevents pathogenic colonization and attenuates inflammatory responses [49]. Conversely, dysbiosis is implicated in the development of various allergic pathologies, with potential long‐term sequelae [50], particularly asthma pathogenesis [51]. Studies have shown that asthma patients are often accompanied by a decrease in the diversity of intestinal microbiota, such as a decrease in the phylum *Bacteroidetes* and an increase in the proportion of *Firmicutes*, especially associated with the depletion of short‐chain fatty acid‐producing bacteria (such as *Rothia* and *Faecalibacterium*) [52]. During gut microbiota dysbiosis, bioactive compounds secreted by microbes and absorbed into the “gut‐lung” axis circulation can directly modulate pulmonary function [53], with each microbial molecular pattern corresponding to a distinct asthma phenotype [5]. Studies reveal that in severe asthma patients, epithelial genomic signatures associated with non‐type 2 inflammatory asthma (marked by Th17 cells) are correlated with aberrantly elevated *Proteobacteria* abundance [17]. Moreover, dysbiosis of the microbiosis can further reduce the level of asthma control and worsen neutrophilic airway inflammation [18, 19]. In a murine model of asthma, Da et al. demonstrated that *Streptococcus boulardii* ameliorates airway inflammation and oxidative stress by restoring gut microbial‐metabolic homeostasis through N6‐methyladenosine (m6A)‐dependent upregulation of methyltransferase‐like 3 (METTL3), thereby attenuating asthma‐induced pulmonary parenchymal injury [54]. The administration of antibiotics and other pharmacological agents significantly disrupts the equilibrium of both gut and pulmonary microbiota. The resultant dysbiosis induces bidirectional crosstalk disruption within the gut‐lung axis, precipitating hypersensitivity reactions through aberrant immune priming and barrier dysfunction [55]. Collectively, alterations in the abundance of specific gut microbial taxa exacerbate the clinical manifestations and inflammatory burden of asthma, while conversely, the pathogenesis and progression of asthma reciprocally manifest as intestinal dysbiosis through the gut‐lung circuit. Table 1 summarizes the key findings of the study of intestinal dysbiosis in patients with asthma.

**TABLE 1:** Overview of research on gut mycobiome in patients with asthma.

Study	Region	Design	Methods	Outcome	Gut mycobiome with outcome
Marie‐Claire Arrietae et al. [40]	Canadian	*n* = 319 with healthy babies	Stool sample collected at age 3 and 12 months	Infants at risk of asthma	Decreased: *Lachnospira*; *Veillonella*; *Faecalibacterium*; *Rothia*
M Demirci et al. [41]	Turkey	*n* = 92 children who were diagnosed with asthma; *n* = 88 with healthy children	Stool sample collected at age 3–8 years old	Physician‐diagnosed asthma	Decreased: *Akkermansia muciniphila* and *Faecalibacterium prausnitzii*
Jakob Stokholm et al. [45]	Danish	*n* = 690 with children at age 12 months	Stool sample collected at age 12 months	Physician‐diagnosed asthma at age 5 years old	Increased: *Veillonella* Decreased: *Roseburia*; *Alistipes*; *Flavonifractor*
Kei E fujimura et al. [46]	Southeastern Michigan, United States	*n* = 298 with children at age 1–11 months	Stool sample collected at age 1–11 months	Physician‐diagnosed asthma at 4 years	Increased: *Candida* and *Rhodotorula* Decreased: *Bifidobacterium*; *Akkermansia* and *Faecalibacterium*
Martin Depner et al. [56]	Rural Austria, Finland, France, Germany, and Switzerland	*n* = 930 with children at age 2–12 months	Stool sample collected at age 2–12 months	Physician‐diagnosed asthma OR parental reports of bronchitis at 6 years	Decreased: *Alternaria*
Amjad N Kanj et al. [57]	United States	*n* = 24 with physician‐diagnosed asthma at age 46–69 years old	Stool sample collected at four time points between age 0–24 months	Severe asthma exacerbation in the past year	Increased: *Candida* to bacterial ratio
Courtney hoskinson et al. [58]	Canada	*n* = 1115 babies	Stool sample collected at age 3 months and 1 years old	Physician‐diagnosed asthma at 5 years	Increased: *Anaerostipes hadrus*; *Fusicatenibacter saccharivorans*; *Eubacterium hallii* and *Blautia wexlerae* Decreased: *Eggerthella lenta*; *Escherichia coli*; *Enterococcus faecalis*; *Clostridium innocuum* and *Tyzzerella nexilis*
Kasper Schei et al. [59]	Norway	*n* = 278 babies	Stool sample collected at four time points between age 0–24 months	Parental report of physician‐diagnosed asthma at 6 years	Increased: Fungal abundance

#### 4.2.2 Th1/Th2/Treg Balance

**FIGURE 2:**
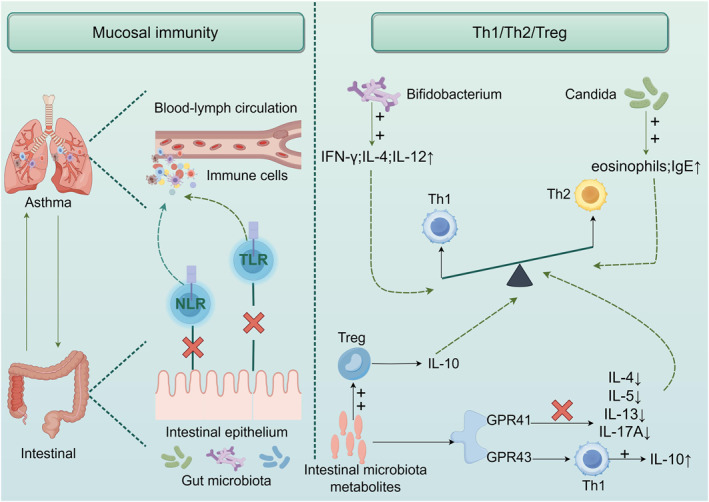
Mucosal immunity and Th1/Th2/Treg. Th1/Th2/Treg balance: *Bifidobacteria* can stimulate Th1/Th2 balance by upregulating inflammatory factors such as IFN‐γ, IL‐4, and IL‐12 in the lungs. Intestinal *Candida* promotes the secretion of IgE and eosinophils, enhances Th2 inflammatory response, and thus regulates Th1/Th2 balance. In addition, metabolites of intestinal microbiota (such as butyrate and propionate) can increase the differentiation of Tregs, promote IL‐10 secretion, and inhibit Th2 cell function. Among them, propionate can reduce the content of Th2‐related pro‐inflammatory cytokines IL‐13, IL‐4, IL‐5 and IL‐17A in the lungs through short‐chain fatty acid receptor GPR41, and can also promote the production of immunosuppressive cytokine IL‐10 by Th1 cells through short‐chain fatty acid receptor GPR43, and finally regulate the balance between Th1 and Th2 responses to regulate the pathogenesis of asthma. Mucosal immunity: The gut microbiota activates the immune system through pattern recognition receptors (PRRs) such as cytoplasmic NOD‐like receptors (NLRs) and membrane‐bound toll‐like receptors (TLRs), influencing the inflammatory response of the lungs through blood‐lymphatic circulation.

Asthma is a chronic inflammatory airway disease characterized by Th2 lymphocyte‐driven pathology, categorized into type 2 and non‐type 2 inflammatory endotypes. A disequilibrium in T‐lymphocyte subset ratios (Th1/Th2) represents a pivotal pathomechanism in asthma development [60, 61]. Asthma is a variable disease here. It may be that some of this variability relates to different diets or environmental/gut exposures although this needs to be confirmed in human studies. Emerging evidence demonstrates that the gut microbiota modulates asthma pathogenesis by regulating the Th1/Th2 response equilibrium [55, 62]. Studies have found that *Bifidobacteria* can stimulate Th1/Th2 balance, upregulate inflammatory factors such as IFN‐γ, IL‐4, and IL‐12 in the lungs, and regulate asthma attacks [63, 64]. It has also been shown that lactic acid bacteria can alter the pathophysiology of asthma by modulating the Th1/Th2 immune response [65]. The study by Amjad et al. demonstrated that mice with gut *Candida* dysbiosis exhibit exacerbated Th2‐mediated responses following airway sensitization with house dust mite (HDM), manifesting as elevated total leukocyte and eosinophil counts in the airways and increased serum total IgE concentrations. The reason for this may be the enhanced Th2 inflammatory response through ILC2 [57]. Notably, oral administration of bacterial lysates to modulate gut microbiota composition resulted in a significant reduction in pulmonary Th2 cell populations [66]. Furthermore, gut microbiota‐derived metabolites, such as butyrate and propionate, promote the differentiation of regulatory T cells (Tregs) [8]. These Tregs suppress Th2 cell functionality via cell‐cell contact or secretion of immunosuppressive cytokines, such as IL‐10 and transforming growth factor‐β(TGF‐β). Concurrently, these metabolites enhance the differentiation and functional capacity of Tregs and Th1 cells by stimulating the cluster of differentiation 4‐positive (CD4+) forkhead box protein P3 (Foxp3) transcriptional axis [67], thereby alleviating allergic hypersensitivity through dual modulation of immune tolerance and Th1/Th2 equilibrium [68]. Among them, propionate can signal through the short‐chain fatty acid receptor G protein‐coupled receptor 41 (GPR41), thereby reducing the content of Th2‐related pro‐inflammatory cytokines IL‐13, IL‐4, IL‐5 and IL‐17A in the lungs [69], and can also promote the production of immunosuppressive cytokine IL‐10 by Th1 cells through the short‐chain fatty acid receptor G protein‐coupled receptor 43 (GPR43) [70], which ultimately prevents allergen‐induced peribronchial inflammation. Airway hyperresponsiveness and airway mucus production. Figure 2 summarizes the mechanism of the Th1/Th2/Treg balance.

#### 4.2.3 IgA and Mucosal Immunity

The crosstalk between the intestinal tract and lungs occurs through lymphatic and circulatory systems, which is critical for transmitting immunological information via the “gut‐lung” axis across organs [29, 71]. The dynamic interplay observed along the “lung‐gut axis” may be associated with shared embryonic origins of gastrointestinal and respiratory mucosal barriers, coupled with functional correlations in physiological and structural aspects [13]. Both systems possess specialized physical barriers featuring microvilli (intestinal epithelium) or ciliated structures (respiratory epithelium), These microvilli are also involved in the local immune response along with lymphoid tissue. Furthermore, both systems share secretory IgA (sIgA) and mucus‐producing goblet cells [72, 73]. The interactions between the host and microbial antigens/metabolites are mediated through the intestinal mucosal barrier formed by a monolayer of epithelial cells [74, 75]. The gut microbiota can stimulate sIgA production, thereby maintaining mucosal barrier integrity and reducing pathogen translocation to the lungs [13]. Additionally, the mucosal linings of both the intestinal and respiratory tracts are enriched with lymphoid tissues and share subsets of immune cells, such as regulatory T cells and dendritic cells. The gut microbiota activates the immune system via pattern recognition receptors (PRRs), including cytosolic NOD‐like receptors (NLRs) and membrane‐bound Toll‐like receptors (TLRs), thereby modulating inflammatory responses in distal organs, such as the lungs [76]. It has been demonstrated in murine models that lipopolysaccharide (LPS) can translocate to the lungs via a compromised intestinal epithelial barrier, where it induces LPS‐induced acute lung injury through activation of the Toll‐like receptor 4 (TLR4)/NF‐κB pathway and subsequent upregulation of pro‐inflammatory cytokines, including IL‐1β, IL‐6, and TNF‐α [77]. Figure 2 summarizes the mechanism of mucosal immunity.

### 4.3 Regulatory Role of Microbial Metabolites

#### 4.3.1 Short‐Chain Fatty Acids (SCFAs)

Short‐chain fatty acids, defined as volatile fatty acids containing fewer than six carbon atoms [70], are microbial fermentation products predominantly comprising acetate, propionate, and butyrate [78]. Acting as immunomodulators, they suppress immune responses by inhibiting chemotaxis and adhesion of immune cells, inducing apoptosis of activated immune cells, and stimulating secretion of anti‐inflammatory cytokines [79]. SCFAs regulate Treg cells differentiation through G protein‐coupled receptors (GPR41/GPR43) expressed on helper T cells or via inhibitory pathways targeting histone deacetylases (HDACs) in cytotoxic T lymphocytes. Subsequently, Treg cells suppress Th2 cell function through cell‐cell contact or secretion of immunosuppressive cytokines such as IL‐10 and transforming growth factor‐β [80], thereby attenuating Th2‐mediated inflammation and playing a pivotal role in preventing allergic airway diseases [81, 82].

**FIGURE 3:**
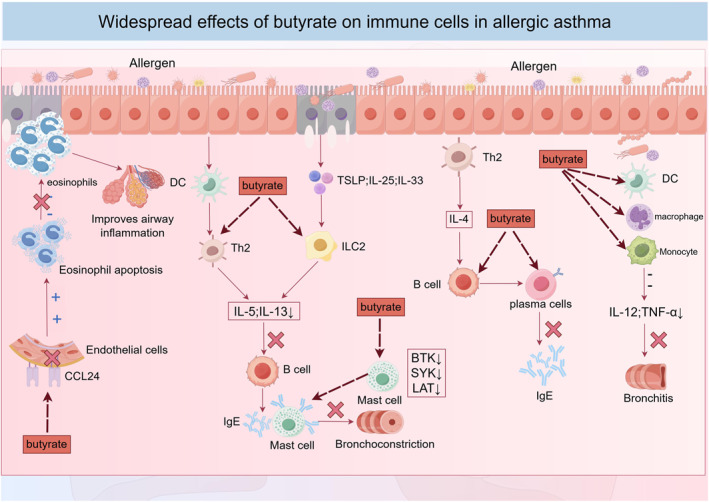
Widespread effects of butyrate on immune cells in allergic asthma. Butyrate promotes eosinophil apoptosis by inhibiting the secretion of IL‐5 and IL‐13 cytokines secreted by Th2 cells and ILC2, and also inhibits the adhesion of eosinophil chemokine‐2 (CCL24) to the vascular endothelium, thereby promoting eosinophil apoptosis and reducing the migration of eosinophils to the lungs, thereby improving airway inflammation. Butyrate can also reduce the expression of transcripts of Bruton's tyrosine kinase (BTK), spleen tyrosine kinase (SYK), and activated T cell linker (LAT) in mast cells, thereby inhibiting IgE‐mediated mast cell degranulation. In addition, butyrate negatively regulates the expression of B cells, leading to a dose‐dependent reduction in plasma cell differentiation, ultimately leading to a decrease in circulating IgE levels. Maturation of dendritic cells, macrophages, and monocytes can also be inhibited by inhibiting the ability of dendritic cells, macrophages, and monocytes to recognize antigens and produce pro‐inflammatory cytokines such as IL‐12 and TNF‐α.

Intestinal‐derived acetate has been demonstrated to protect against influenza‐induced pulmonary injury by maintaining airway epithelial integrity and mucosal homeostasis [83]. Propionate, as evidenced by in vivo and in vitro experimental models in mice, enhances the expression of IL‐10 and the Treg‐specific transcription factor Foxp3, thereby attenuating inflammatory responses [84]. Furthermore, studies have shown that propionate and butyrate administration in murine models of allergic asthma significantly reduces eosinophil activity and suppresses airway inflammation [8]. Butyrate ameliorates allergic asthma by modulating distinct pathways across immune cell compartments [85]. Specifically, butyrate suppresses the secretion of IL‐5 and IL‐13 cytokines by Th2 cells and type 2 innate lymphoid cells, while concurrently inhibiting eosinophil chemotactic factor‐2 (eotaxin‐2/CCL24)‐mediated adhesion to vascular endothelium [86]. These mechanisms collectively promote eosinophil apoptosis and attenuate eosinophil migration to pulmonary tissues, thereby alleviating airway inflammatory responses [87]. Butyrate downregulates the expression of transcripts encoding Bruton's tyrosine kinase (BTK), spleen tyrosine kinase (SYK), and linker for activation of T cells (LAT) in mast cells, thereby suppressing IgE‐mediated mast cell degranulation [88]. Studies have demonstrated that butyrate negatively regulates the expression of activation‐induced cytidine deaminase (Aicda) and B lymphocyte‐induced maturation protein 1 (Blimp‐1) in murine and human B cells, resulting in a dose‐dependent reduction in plasma cell differentiation and suppression of class‐switch recombination to IgG, IgA, and IgE, ultimately leading to decreased circulating IgE levels [89]. Furthermore, butyrate inhibits the maturation of dendritic cells, macrophages, and monocytes by impairing their capacity for antigen recognition and production of pro‐inflammatory cytokines such as IL‐12 and TNF‐α [90]. Figure 3 summarizes the Effect of butyrine hydrochloric acid on immune cells in allergic asthma.

#### 4.3.2 Bile Acids

Primary bile acids, synthesized via hepatic biotransformation of cholesterol, undergo structural modifications by the gut microbiota to generate secondary bile acids, which exert biological effects by modulating gut microbial homeostasis [91]. Studies have demonstrated that secondary bile acids activate the cAMP/protein kinase A (PKA) signaling cascade through G protein‐coupled receptor‐mediated pathways. This mechanism suppresses pro‐inflammatory cytokine expression by inhibiting monocyte‐derived dendritic cell activation and nuclear factor‐kappa B (NF‐κB) signaling, ultimately mediating bronchial smooth muscle relaxation and ameliorating airway hyperresponsiveness [92]. Studies have also demonstrated that conjugated bile acids (such as tauro‐ursodeoxycholic acid, alanyl‐β‐muricholic acid) bind to activating transcription factor 6α(ATF6α), subsequently attenuating allergen‐induced activation of the unfolded protein response. This mechanism significantly reduces inflammation, immune and cytokine responses, mucous metaplasia, and airway hyperresponsiveness [93]. The farnesoid X receptor (FXR), encoded by the Nr1h4 gene [94], functions as a bile acid receptor and exerts anti‐inflammatory effects in ovalbumin‐induced rat asthma models. This is achieved by suppressing the secretion of IL‐4, IL‐5, and IL‐13 from activated helper Th2 cells, as well as TNF‐αfrom activated alveolar macrophages, thereby blocking inflammatory cell infiltration into healthy lung tissue [95].

#### 4.3.3 Tryptophan Metabolites

Beyond short‐chain fatty acids and bile acids, other bacterial metabolites also contribute to immune regulation in asthma. For instance, tryptophan metabolites derived from the gut microbiota act as ligands for the aryl hydrocarbon receptor (AhR) [96], thereby facilitating immunomodulation. Specific tryptophan catabolites, such as kynurenine (KYN), indole‐3‐lactic acid (ILA), indole‐3‐carboxaldehyde (I3C), and indole acetic acid (IAA), exert their effects on asthma pathogenesis through distinct molecular mechanisms. Preclinical studies have demonstrated that I3C ameliorates pulmonary inflammation in asthmatic mice by significantly reducing serum levels of OVA‐sIgE and downregulating inflammatory cytokines, including IL‐10 and IL‐17, in bronchoalveolar lavage fluid. In contrast, KYN alleviates OVA‐induced allergic asthma by restoring gut microbial diversity. Furthermore, ILA and I3C may promote a Th2‐to‐Th1 phenotypic shift, thereby attenuating type 2‐driven inflammatory responses in asthma [97]. Additionally, IAA binds to aryl hydrocarbon receptor ligands, such as indole‐3‐carboxaldehyde, and promotes airway epithelial barrier repair by modulating IL‐22 expression [98]. Furthermore, other gut microbiota‐derived metabolites, including indole derivatives, polyamines, and deaminated tyrosine, exhibit immunomodulatory properties; however, their specific roles in asthma pathogenesis require further investigation [99].

### 4.4 Neuro‐Immune Regulatory Pathways

An additional critical pathway within the “gut‐lung” axis involves neuroactive mediators, including neurotransmitters and neuropeptides, produced by the enteric nervous system in the gut. These signaling molecules modulate respiratory system activity via vagus nerve‐mediated “gut‐lung” communication, regulating physiological processes such as immune responses, inflammation, and pulmonary functional coordination [100]. For instance, gut microbiota‐derived metabolites influence pulmonary inflammation by engaging the vagus nerve to activate the cholinergic anti‐inflammatory pathway, thereby attenuating airway inflammation [101]. Additionally, vasoactive intestinal peptide (VIP), a neuropeptide secreted by both pulmonary and colonic tissues [102], is widely distributed in the gut and exerts potent bronchodilatory effects on airways [103]. Research indicates that gut microbiota‐derived butyrate activates G protein‐coupled receptors (GPR41/43) on enterochromaffin cells, thereby inducing the release of 5‐hydroxytryptamine (5‐HT). This 5‐HT subsequently activates pulmonary 5‐HT receptors, promoting smooth muscle contraction and exacerbating asthmatic symptoms [79]. However, the molecular mechanisms underlying how neuro‐immune crosstalk within the “gut‐lung” axis drives asthma pathogenesis remain incompletely elucidated, necessitating further experimental investigations to unravel these pathways.

## 5 Asthma Treatment Strategies That Target the Enteropulmonary Circulation

### 5.1 Probiotics and Prebiotics

Probiotics and prebiotics, classified as indigestible dietary fibers primarily derived from the intestinal microbiota of healthy individuals, represent distinct biological entities. Probiotics refer to viable microbial strains conferring health benefits, whereas prebiotics constitute growth‐promoting substrates that selectively stimulate the proliferation of probiotics and other beneficial microbial communities [104]. With advancing theoretical elucidation of the “gut‐lung” axis regulatory mechanisms, probiotics and prebiotics have emerged as promising therapeutic modalities for both asthma prophylaxis and clinical management.

#### 5.1.1 Probiotics

Extensive research demonstrates that both single and combined probiotic strains can modulate intestinal mucosal maturation and immunotolerance, influence dendritic cell ontogeny, and regulate systemic immune responses, ultimately exerting significant therapeutic effects against asthma [105]. Preclinical validation in murine models has established lactobacilli as the most promising probiotic candidates for asthma prevention and management. Administration of *Lactobacillus fermentum* in asthmatic mice significantly ameliorates pulmonary inflammation and fibrotic remodeling, while concurrently suppressing pulmonary expression levels of pro‐inflammatory mediators including IL‐4, IL‐5, and IL‐13 in lung tissue [65]. Emerging evidence indicates that oral administration of *Lactobacillus reuteri* enhances splenic CD4(+)CD25(+)Foxp3(+) regulatory T cell populations and functional activity, while attenuating OVA‐induced allergic airway inflammation in sensitized murine models [106]. An experimental study revealed that therapeutic intervention with *Lactobacillus rhamnosus* in OVA‐sensitized mice suppressed methacholine‐evoked airway hyperresponsiveness, significantly reducing inflammatory cell infiltration and Th2 cytokine concentrations in bronchoalveolar lavage fluid and serum. Furthermore, this probiotic intervention demonstrated immunomodulatory efficacy through downregulation of OVA‐specific IgE titers in serum, concomitant suppression of matrix metalloproteinase‐9 (MMP‐9) expression in pulmonary tissue, and inhibition of inflammatory cell transmigration [107]. Mixed probiotic treatment can also improve asthma, and one animal experiment showed that multi‐strain probiotic formulation (*Lactobacillus gasseri*, *Lactobacillus salivarius*, *Lactobacillus johnsonii*, *Lactobacillus paracasei*, *Lactobacillus reuteri*, *Bifidobacterium* animals) in murine asthma models significantly attenuates OVA‐induced allergic airway inflammation via coordinated downregulation of key pro‐inflammatory mediators, including IL‐4, IL‐13, IL‐17A, eosinophil cationic protein, and neutrophil elastase levels; It also inhibits airway remodeling characteristics in asthma mice. This mechanism may be to inhibit allergic asthma by mesenteric CD103+DCs inducing mucosal initiation of regulatory T cell differentiation and inhibition of Th2 allergic responses [108]. A clinical investigation by Huang et al. demonstrated that both *Lactobacillus paracasei* and *Lactobacillus fermentum* monotherapies effectively reduce asthma severity scores and enhance Asthma Control Test outcomes in school‐aged pediatric cohorts. Notably, the combination therapy exhibited synergistic immunomodulatory effects, a combination of two probiotics is more effective than using one or the other alone [8].

#### 5.1.2 Prebiotics

Beyond probiotic interventions, prebiotic supplementation demonstrates therapeutic potential in asthma pathogenesis modulation. Experimental research in murine models revealed that administration of prebiotics (fructooligosaccharides and galactooligosaccharides) significantly attenuates pulmonary recruitment of activated eosinophils while suppressing eosinophil peroxidase (EPO) activity in bronchoalveolar lavage fluid. This intervention concurrently reduces BALF concentrations of immunoglobulins, IL‐17, and guanosine triphosphate (GTP), while histopathologically ameliorating mucus hypersecretion, goblet cell hyperplasia, and peribronchial/perivascular inflammatory infiltrates, thereby modulating key pathways in asthma development [109].

#### 5.1.3 The Timing and Methods of Using Probiotics and Prebiotics

At present, there is no consensus on the recommendation of probiotic dosage, and some experiments have proved that there is an optimal time point and use mode of probiotic application. Oral administration is currently the standard route for probiotics, but Céline et al.'s study showed that intranasal administration of *Lactobacillus paracasei* was more effective than oral administration in modulating allergic airway inflammation in mice [110]. In the intestinal microbiota, when probiotics are used in the neonatal period, the abundance of firmicutes and actinobacteria increases, accompanied by an increase in the number of CD4 Treg cells and butyrate in alveolar lavage fluid; When probiotics were applied to adult animals, only Actinobacteria were significantly more abundant than those in the control mouse [111]. A meta‐analysis of clinical trials showed that probiotic treatment during pregnancy and/or the first few years of life did not reduce the risk of asthma or wheezing [112]. Similarly, another meta‐analysis of 19 randomized controlled trials showed that treating infants with probiotics was not associated with a reduced risk of asthma [113]. Current consensus is lacking regarding optimal administration protocols for probiotic interventions in asthma management, including dosage regimens, initiation timing and duration, delivery modalities, and patient population stratification. Future research is required to validate the preventive efficacy of probiotics against asthma development and establish evidence‐based guidelines for both asthma prophylaxis and probiotic therapeutic implementation to guide clinical practice.

### 5.2 Dietary Interventions

The gut microbiota exhibits diet‐induced compositional plasticity. Consequently, scientific inquiry has focused on whether dietary modulation impacts pulmonary and intestinal microbial ecosystems via the “gut‐lung” axis, thereby influencing asthmatic airway inflammation, clinical manifestations, and disease progression. Elevated intestinal concentrations of short‐chain fatty acids demonstrate significant positive correlations with improved spirometric parameters, suggesting dietary fiber interventions may confer prophylactic effects against inflammatory airway diseases through microbiota‐host metabolic crosstalk [29]. Current research data indicate that dietary patterns modulate airway inflammatory responses through homeostatic regulation of gut microbiota‐derived metabolites (such as short‐chain fatty acids), mediated by pro‐/anti‐inflammatory cytokine networks, establishing a bidirectional regulatory axis between the gastrointestinal tract and respiratory system. This complex crosstalk network may provide novel insights into asthma therapeutics [81].

A pivotal connection between diet and the gut‐lung axis involves dietary fiber consumption, particularly from fruits, vegetables, whole grains, and legumes. This intervention prevents asthma through enhancing epithelial barrier integrity, promoting Treg induction, attenuating Th2 polarization, and suppressing mast cell overactivation, thereby counteracting allergen‐induced peribronchial inflammation, airway hyperresponsiveness, and mucus hypersecretion [114]. Research demonstrates that dietary supplementation with omega‐3 fatty acids modulates alveolar macrophage polarization phenotypes and neutrophil extracellular trap formation, optimizing innate immune defense mechanisms. This intervention effectively curtails inflammatory cascades and pulmonary tissue remodeling, thereby mitigating both the risk of asthma development and disease severity [115]. A study demonstrates that in adults with severe asthma, dietary fiber intake exhibits an inverse correlation with airway eosinophilia and lung function decline [116]. Furthermore, adult dietary fiber consumption modulates inflammatory responses, reduces asthma incidence, and prevents the onset of cough, wheezing, and sputum production [117].

In murine models, high‐fat diet consumption exacerbates allergic airway inflammation and ozone‐induced airway hyperresponsiveness [118]. Research indicates that Western dietary patterns—characterized by ultra‐processed food consumption and high‐frequency fast‐food intake with insufficient fruit/vegetable and whole‐grain consumption—induce dysregulated macronutrient profiles (elevated saturated fats, refined carbohydrates, sodium intake with micronutrient deficiencies) and insufficient dietary fiber, vitamins, and phytobioactive compounds. This metabolic‐inflammatory axis dysregulation may drive elevated asthma incidence through mechanisms including intestinal immune microenvironment remodeling and exacerbated oxidative stress responses [119]. Olive oil, a principal component of the Mediterranean diet enriched with polyphenols and fatty acids, demonstrates correlation with improved asthma control. Collectively, high‐fiber dietary patterns (such as Mediterranean diet) enhance production of beneficial metabolites that potentiate anti‐inflammatory immune responses, maintain epithelial barrier integrity, and attenuate asthma severity. In contrast, Western‐style diets characterized by high‐fat/low‐fiber intake disrupt gut microbial homeostasis and upregulate pro‐inflammatory metabolites, exacerbating airway inflammation and asthma symptomatology.

Certain antioxidants, particularly polyphenols, modulate gut microbial composition. For instance, polyphenols from green tea, grapes, and cocoa enhance proliferation of beneficial bacteria such as *Lactobacillus* and *Bifidobacterium*, thereby regulating immune system function [120]. Research by Esraah et al. demonstrates that the phytopolyphenol resveratrol alleviates asthma via the gut‐lung axis by inducing beneficial microbiota and enhancing pulmonary barrier integrity. Specifically, resveratrol significantly elevates the relative abundance of *Bacteroidetes*, *Bacteroidales*, and *Bacteroides acidifaciens* in OVA‐induced asthmatic murine models, modulating gut microbiota composition. Concurrently, it upregulates pulmonary epithelial tight junction proteins while suppressing mucin overproduction, thereby reinforcing pulmonary barrier architecture [121]. Dietary polyphenols exhibit high binding affinity with food proteins, forming soluble and insoluble protein‐phenolic complexes that reduce food allergenicity. Specifically, polyphenol‐allergen interactions (via covalent/non‐covalent binding) induce conformational structural modifications in allergenic proteins, thereby diminishing IgE‐binding capacity—a critical determinant of allergenic potential [122]; different polyphenols have been found to mask the linear epitope of the allergen by binding to nucleophilic amino acids, and to alter the conformational epitope of the allergen by altering the secondary and tertiary structure of the protein, thereby reducing the allergen's sensitization [123].

Research demonstrates that vitamin D exerts crucial immunomodulatory effects on gastrointestinal and pulmonary health by enhancing intestinal barrier integrity, mitigating bacterial translocation and endotoxin dissemination into systemic circulation, attenuating systemic/pulmonary inflammation, reducing asthma exacerbations, and improving pulmonary function parameters [124]. Research by Hanna et al. revealed significant associations between maternal hypovitaminosis D during gestation and pediatric asthma incidence, as well as impaired pulmonary function in high‐risk pediatric cohorts, and suggested that a gestational 25(OH)D level of ≥ 30 ng/mL may be most appropriate for respiratory health in the offspring of high‐risk subjects with asthma, but further studies are needed to determine the optimal pregnancy 25(OH)D level in the general population [125]. A randomized clinical trial by Erick et al. involving 192 pediatric subjects demonstrated that vitamin D3 supplementation did not significantly prolong the time‐to‐severe asthma exacerbation. Similarly [126], Anne et al.'s study encompassing pediatric and adult populations with asthma revealed that supplemental vitamin D or its hydroxylated metabolites demonstrated no therapeutic efficacy in reducing asthma exacerbation risk or improving disease control metrics [127]. At present, there is no clear consensus between the molecular mechanism and clinical benefit of vitamin D in improving asthma through immunomodulatory effects, and its anti‐inflammatory pathway regulatory effect and individualized dosing regimen need to be verified by evidence‐based medicine through large‐sample randomized controlled trials combined with dynamic monitoring of biomarkers. Table 2 summarizes the effects of various dietary patterns or components on the gut microbiota and asthma.

**TABLE 2:** The effects of various dietary patterns or ingredients on the gut microbiota and asthma.

Dietary pattern/ingredients	Characteristics	Impact on gut microbiota	Impact on asthma
High‐fiber diet	Especially high‐fiber foods from fruits, vegetables, whole grains, and legumes	Promote the production of beneficial metabolites and maintain the balance of intestinal microbiota	Strengthens the epithelial barrier, promotes treg cell induction, alleviates Th2 polarization and inhibits mast cell hypersecretion, prevents allergen‐induced peribronchial inflammation, airway hyperresponsiveness and airway mucus production, and thus prevents asthma
Western diet	Deeply processed foods and high‐frequency fast food, which are high in saturated fat, high in carbohydrates, and accompanied by trace element deficiencies	Disrupts the balance of intestinal microbiota and upregulates pro‐inflammatory metabolites	Mechanisms such as enhancing airway pro‐inflammatory immune response, reshaping the intestinal immune microenvironment, and exacerbating oxidative stress increase the frequency and severity of asthma attacks
Mediterranean diet	It is characterized by an intake of olive oil, vegetables, fruits, whole grains, legumes and fish, low intake of saturated fats and processed foods	Increase microbial diversity and maintain intestinal microecological balance	Promotes the production of beneficial metabolites to increase anti‐inflammatory, immune responses, maintain epithelial barrier integrity, and reduce asthma severity
Rich in polyphenols	It is characterized by the intake of green tea, grapes, and cocoa, among others	Promotes the growth of beneficial bacteria such as lactobacilli and bifidobacteria, which regulates the immune system	Induces beneficial microbiota and promotes normal barrier function of the lungs, reducing allergen sensitization and thereby alleviating asthma
Vitamin D	Vitamin D supplements or their hydroxylated metabolites	Strengthens intestinal barrier function, reduces the translocation of harmful bacteria and endotoxins into the bloodstream	Reducing systemic and lung inflammation, reducing asthma attacks and improving overall lung function is controversial

### 5.3 Fecal Microbiota Transplantation (FMT)

Fecal microbiota transplantation involves transferring fecal material containing intact and stable gut microbial communities from healthy donors to recipients, aiming to restore gut microbiota equilibrium and modulate immune function [128]. Clinical practice has confirmed its remarkable therapeutic efficacy in intestinal disorders, with the deepening of the “gut‐lung” axis theory, FMT may have significant therapeutic potential for asthma.

Studies have reported that transplanting *Bacteroides fragilis*‐enriched fecal microbiota from adult asthma patients into murine models induces Th17‐mediated inflammatory responses and oxidative stress in the airways [129]. Imbalances in gut microbiota and short‐chain fatty acids constitute a critical mechanism underlying asthma recurrence. FMT therapy ameliorates inflammatory responses in asthmatic animal models by promoting intestinal SCFA restoration and facilitating gut microbiota remodeling to alleviate asthma symptoms. Studies by Yitian et al. demonstrated that, compared to the asthmatic rat group, fecal‐transplanted asthmatic rats exhibited significantly improved pulmonary function, reduced lymphocyte and eosinophil counts in peripheral blood and bronchoalveolar lavage fluid, and markedly decreased protein and mRNA levels of inflammatory cytokines (IL‐4, IL‐5, IL‐13, IL‐17, IL‐33) and mediators (LT, TSLP, PGD2) in lung tissue, alongside alleviated histopathological alterations and collagen fiber deposition; additionally, FMT elevated propionate, butyrate, and pentetohydrochloride levels [130]. Similarly, studies by Wu et al. demonstrated that fecal microbiota transplantation from donors fed with curcumin (Cur) or its primary active metabolite tetrahydrocurcumin (THC) into OVA‐induced asthmatic mice revealed that both Cur and THC treatments altered gut microbiota composition in asthmatic mice, characterized by a significant reduction in the *Firmicutes* to *Bacteroidetes* ratio; additionally, they decreased the relative abundance of pro‐inflammatory bacteria such as *Proteobacteria*, *Intestinimonas*, Unidentified‐*Ruminococcaceae*, and *Lachnospiraceae*, thereby ameliorating asthma [131]. Currently, evidence‐based medical evidence for fecal microbiota transplantation in asthma treatment remains insufficient, and the long‐term efficacy evaluation and biosafety validation still require systematic verification through multicenter, large‐sample prospective cohort studies.

### 5.4 Helminth Therapy

Allergic asthma is a classic immune‐mediated inflammatory disease. Given the immunomodulatory properties of helminths or helminth‐derived molecules, helminth therapy also holds potential as another avenue for asthma prevention and treatment. Existing studies indicate that helminth infection reduces the incidence of allergic asthma, as helminths typically induce protective host type 2 cell‐mediated immunity, thereby limiting type 1 inflammation, reducing host tissue damage, and alleviating asthmatic inflammatory responses [132].

Helminth infection modulates immune responses by regulating distinct inflammatory cells and cytokines; it induces damaged intestinal epithelial cells to produce IL‐25, IL‐33, and TSLP, further activating and recruiting ILC2s and Th2 cells. These cells secrete type 2 cytokines (IL‐4, IL‐5, IL‐10, IL‐13) and GM‐CSF, thereby promoting eosinophilia, M2 macrophage polarization, and secretion of IgG1, IgG4, and IgE, amplifying Th2 immune responses while suppressing Th1 immune responses [133]; IL‐4 stimulates B cells to produce helminth‐specific IgE, which opsonizes helminths and binds to IL‐5‐activated eosinophils [134]; additionally, intestinal tuft cells secrete cysteinyl leukotrienes during helminth infection, which cooperates with IL‐25 to activate ILC2s to secrete IL‐13, thereby amplifying Th2 immune responses and leading to hyperplasia of mucus‐secreting goblet cells and hypercontraction of smooth muscle [135]. Studies have demonstrated that helminth‐secreted peroxiredoxin induces alternative activation of macrophages and activates naïve T cells (Th0) to secrete IL‐4, IL‐5, and IL‐13 [136]; it also induces Tregs and regulatory macrophages to produce anti‐inflammatory mediators such as TGF‐β, IL‐10, and PGE2 to ameliorate inflammatory responses [137], or activates dendritic cells to drive naïve CD4+ T cell differentiation into Th2 cells in vitro, thereby promoting Th2 immune responses [138].

However, not all helminth infections improve asthma outcomes, as this depends on helminth species and population characteristics across regions. For instance, *Ancylostoma duodenale*, hookworm, *Trichuris trichiura*, *Ascaris lumbricoides*, *Toxocara canis*, and *Necator americanus* confer no protective effects against allergic asthma. A clinical trial by Feary et al. demonstrated that hookworm infection in asthma patients did not lead to significant improvements in bronchial hyperreactivity or other asthma control parameters compared to controls [139].

**FIGURE 4:**
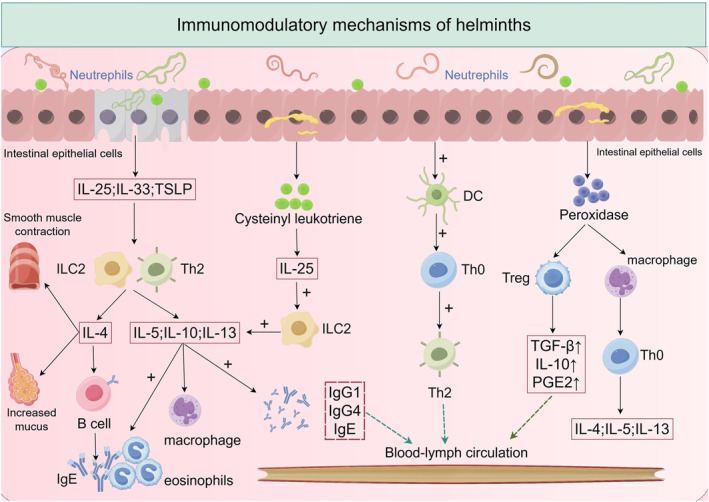
Immunomodulatory mechanisms in helminths. Helminth infection can participate in the immune response by regulating different inflammatory cells and factors; It can further activate and recruit ILC2s and Th2 by inducing the production of IL‐25, IL‐33 and TSLP in broken intestinal epithelial cells, and these cells produce type 2 cytokines (IL‐4, IL‐5, IL‐10, and IL‐13), thereby inducing eosinophilia, M2 macrophage polarization, and IgE secretion to amplify the Th2‐type immune response, thereby inhibiting the Th1‐type immune response. Among them, IL‐4 stimulates B cells to produce worm‐specific IgE and bind to IL‐5‐activated eosinophils; In addition, intestinal cluster cells secrete cysteinyl leukotrienes during helminth infection, which cooperates with IL‐25 to activate ILC2 secretion of IL‐13 and thus amplify the Th2‐type immune response. Peroxide‐reducing proteins secreted by worms induce alternative activation of macrophages and activation of Th0 cells to secrete IL‐4, IL‐5, and IL‐13; It can also induce Treg cells and regulatory macrophages to produce anti‐inflammatory mediators such as TGF‐β, IL‐10, and PGE2 to improve inflammatory responses, or activate DCs to induce Th0 cells to differentiate into Th2 cells in vitro, thereby inducing Th2‐type immune responses.

Current research on helminthic therapy for asthma management remains predominantly confined to preclinical animal studies, with its translational application into clinical medicine facing significant challenges. Specifically, optimal parasitic inoculum concentration thresholds and therapeutic intervention duration parameters for human application have not been conclusively established. The clinical translation of this therapeutic strategy faces three main obstacles: first, the possible toxic side effects caused by the active ingredient of the parasite limit the clinical trials; Secondly, the differences in individual immune responses caused by genetic diversity in the subject population significantly increased the difficulty of analyzing the experimental data. In addition, there is a lack of a standardized biological dose evaluation system, which makes it difficult to establish an equivalent dose conversion relationship between animal models and human applications. Together, these factors have led to controversy over the use of helminths in the treatment of asthma. The immunomodulatory pathways of helminths are shown in Figure 4.

### 5.5 Other Treatments

The study by Shao et al. demonstrated that alanyl‐glutamine ameliorates airway inflammation in asthmatic mice by modulating the microbiota and derived metabolites, through mechanisms involving suppression of the NF‐κB and STAT3 pathways via the gut microbiota‐butyrate‐GPR43 axis, which significantly reduced *Bacteroidetes* and increased *Firmicutes* abundance while elevating intestinal microbial metabolite butyrate levels, thereby reducing cytokines and attenuating inflammatory infiltration in OVA‐induced allergic asthmatic mice [140]. The study by Michelle et al. demonstrated that nitro‐oleic acid (NO2‐OA) reduces hepatic bile acid levels in mice via modulation of bile acid biosynthetic enzyme expression, concurrently decreasing small airway resistance and tissue elasticity, thereby attenuating allergen‐ and obesity‐induced pulmonary dysfunction in murine asthma models [114]. The study by He et al. demonstrated that the traditional Chinese herbal formulation Shaoyao‐Gancao Tang alleviates pulmonary airway remodeling in ovalbumin‐induced asthmatic rats by modulating the Th1/Th2 ratio in lung and intestinal tissues and altering gut microbiota composition. Specifically, the decoction reduced IgE levels in bronchoalveolar lavage fluid and serum, ameliorated inflammatory cell infiltration and goblet cell metaplasia in the lungs, significantly decreased IL‐4 while increasing IFN‐γ levels in pulmonary and colonic tissues, thereby restoring the IFN‐γ/IL‐4 ratio. Additionally, it reduced the abundance of Ethanoligenens and Harryflintia, while elevating the abundance of Ruminococcaceae_UCG‐005 and Candidatus_Sacchrimonas [141]. The study by Kyu et al. demonstrated that the AhR agonist FICZ (6‐formylindolo[3,2‐b]carbazole) significantly reduces pulmonary eosinophilia, serum total IgE and OVA‐specific IgE concentrations, and Th2 cytokine expression in the lungs of OVA‐sensitized and challenged mice. Additionally, the number of IL‐4‐producing T cells in mediastinal lymph nodes was diminished in FICZ‐treated mice, thereby ameliorating asthma [142].

## 6 Animal Models of Gut‐Lung Axis in Asthma

In recent years, the significance of the gut microbiota and its immunomodulatory effects via the “gut‐lung axis” in the pathogenesis of asthma has garnered increasing attention. Currently, although human clinical and observational studies have preliminarily revealed correlations between bidirectional gut‐lung interactions and the development and progression of asthma, the in‐depth elucidation of causal relationships and specific molecular mechanisms still largely relies on animal models. In asthmatic animal models, the classic protocol utilizing ovalbumin as an allergen, combined with adjuvant intraperitoneal sensitization followed by airway nebulization challenge, can faithfully recapitulate core pathological features of asthma, including eosinophilic infiltration, airway hyperresponsiveness, and a Th2‐biased immune response [143]. On the other hand, House Dust Mite (HDM), as a major allergen in human asthma, induces immune responses in constructed models that more closely resemble features of the human disease, including a stronger Th2 inflammation and significant airway remodeling [144]. To investigate the impact of gut microbiota on asthma, researchers commonly employ interventions or disruptions of the intestinal microenvironment in these established asthmatic models. These include antibiotic‐induced microbiota depletion models, dietary fiber intervention models, probiotic/prebiotic intervention models, and fecal microbiota transplantation models. Table 3 summarizes various animal model experiments exploring the association between the gut‐lung axis and bronchial asthma.

**TABLE 3:** Animal models of gut‐lung axis in asthma.

Study	Types of animal models	Induction/intervention methods	Main findings	Main applications
Amjad N Kanj et al. [57]	HDM + antibiotic	Cefoperazone + HDM	Mice with dysbiotic microbiota exhibit enhanced Th2 responses after HDM airway sensitization, worsening asthma control and increasing allergic airway inflammation	Demonstrate the impact of microbiome depletion on the severity of asthma
Emily M Nakada et al. [93]	HDM + bile acid metabolites	HDM + Tauroursodeoxycholic acid	Bile acids can improve inflammation and unfolded protein response in asthma	Prove the effects of microbial metabolites on asthma
Firdose Begum Shaik et al. [95]	OVA + farnesoid X receptor	OVA + Chenodeoxycholic acid	Bile acid receptors can reduce the degree of airway inflammation in asthmatic mice	Proving the effect of microbial metabolites' receptors on asthma
Hongchao Wang et al. [97]	OVA + tryptophan metabolites	OVA + KYN、ILA、I3C and IAA	Tryptophan metabolites improved the survival and asthma symptoms of asthmatic mice, and reduced inflammatory cells in lung tissue	Evidence suggests that tryptophan metabolites may regulate asthma through the gut microbiota
Da Liu et al. [54]	OVA + probiotic supplements	OVA + *Saccharomyces boulardii*	Probiotics improve inflammation and oxidative stress in asthmatic mice, alleviating lung injury. In addition, they remodel the gut microbiota homeostasis of asthmatic mice and restore their metabolic balance	Prove the effects of probiotics on asthma
Esraah Alharris et al. [121]	OVA + plant‐based polyphenol	OVA + resveratrol	Polyphenols can alleviate asthma by modulating beneficial microbiota in the ‘gut‐lung’ axis and promoting normal barrier function in the lungs	Prove the impact of dietary components on asthma
Naomi G Wilson et al. [129]	OVA + fecal microbiota transplantation	OVA + fecal microbiota transplantation in asthma patients	Transplanting feces from asthma patients into mice increases oxidative stress in the lungs and exacerbates airway inflammation	Proving the effects of fecal microbiota transplantation on asthma
Hermelijn H Smits et al. [145]	OVA + worm infection	OVA + *Schistosoma mansoni*	Chronic and intense worm infections may suppress allergic airway inflammation in asthma	Prove the effect of worm infection on asthma
Sugan Qiu et al. [146]	HDM + worm infection	HDM + *Schistosoma japonicum*	Worm infection reduces airway hyperresponsiveness in asthma and improves airway inflammation	Prove the effect of worm infection on asthma

## 7 Biomarkers and Diagnostic Indicators

The diagnosis and phenotypic classification of asthma have long relied on clinical manifestations, pulmonary function tests, and conventional inflammatory markers. However, these parameters often fail to fully reflect the disease's heterogeneity and underlying mechanisms. The evolving concept of the “gut‐lung” axis has revealed that the gut microbiota and its metabolites profoundly influence asthma susceptibility, severity, and treatment response by modulating systemic and local immunity. Consequently, identifying biomarkers related to this axis provides new opportunities for achieving precision medicine in asthma.

As mentioned earlier, 16S rRNA gene sequencing and metagenomic analyses indicate that asthmatic patients exhibit dysbiosis of the gut microbiota; therefore, specific microbial signatures may serve as early biomarkers for predicting asthma risk. Future studies are needed to establish distinct microbial profiles corresponding to different asthma phenotypes. Furthermore, the levels of microbial metabolites, such as short‐chain fatty acids, bile acids, and tryptophan metabolites, are associated with increased asthma risk and disease activity. Among these, bile acids modified by the gut microbiota can regulate immunity via the farnesoid X receptor coupled bile acid signaling [95], and their compositional changes may be linked to asthma. Tryptophan metabolites derived from the gut microbiota can act as ligands for the aryl hydrocarbon receptor [96], contributing to immune regulation, and imbalances in this metabolic pathway may represent a potential biomarker for asthma. Gut‐derived immune signals shape systemic immune status. In asthmatic patients, gut dysbiosis can disrupt the Th1/Th2 cytokine balance, creating a characteristic cytokine profile that may serve as an indicator of disease activity. Although research on gut‐lung axis‐related biomarkers holds great promise, their clinical translation faces numerous challenges. Asthma phenotype, host genetic background, age, diet, and medication use (particularly antibiotics and corticosteroids) significantly influence biomarker levels, necessitating analyses in large, stratified populations. Future research will tend to integrate metagenomics, metabolomics, and immunology data to construct multi‐modal biomarker panels for more accurate prediction of disease risk, phenotyping, and treatment response.

## 8 Integrating Metagenomics and Multi‐Omics Strategies: Deciphering the Molecular Mechanisms of the Gut‐Lung Axis in Bronchial Asthma

The pathogenesis of bronchial asthma involves a complex interaction between genetic predisposition and environmental factors. The “gut‐lung” axis provides a novel paradigm for understanding this heterogeneity, emphasizing that gut microbiota and their metabolites remotely regulate pulmonary inflammation through immune, neural, and endocrine pathways. However, deciphering this complex interaction network requires moving beyond descriptions of microbial composition to deeply investigate its functional outputs and effects on the host. Metagenomics and integrated multi‐omics analyses provide powerful tools for systematically elucidating the functional role of gut microbiota in asthma. Furthermore, asthma endotyping based on multi‐omics features, by integrating metagenomic and multi‐omics studies, can guide personalized strategies involving probiotics, prebiotics, or dietary interventions, and identify biomarker panels applicable for clinical diagnosis, prognostic prediction, and therapeutic guidance. Elucidating the molecular network of microbe‐host interactions will ultimately advance molecular subtyping and precision therapy for asthma.

### 8.1 Metagenomics: From Species Identification to Functional Potential Analysis

16S rRNA gene sequencing serves as a preliminary screening tool in metagenomics, enabling the assessment of structural dysbiosis in the gut microbiota by analyzing hypervariable regions, thereby further elucidating alterations in the gut microbiota of asthma patients. In contrast, shotgun metagenomic sequencing allows for unbiased sequencing of all microbial DNA in a sample, more directly revealing the functional gene profiles of microbial communities. It can predict the functional potential of the microbiota in key immunomodulatory pathways such as short‐chain fatty acid synthesis, tryptophan metabolism, and bile acid transformation, directly linking specific microbial gene features to asthma phenotypes.

Studies suggest that analyzing the gut microbiome of asthma and obese patients stratified by atopic status or Th2 inflammatory gene signatures may reveal important differences in how the gut microbiome influences clinical outcomes of specific asthma phenotypes. Research by David et al. indicated that the degree of disease inflammation in obese asthma patients was inversely correlated with fecal *Akkermansia muciniphila* levels, while administration of *Aspergillus muciniphilus* to mouse models significantly reduced airway hyperresponsiveness and airway inflammation [147]. Arrieta et al. classified asthmatic children into four clinical phenotypes: atopic + wheeze, atopic only, wheeze only, and control. Using 16S rRNA gene sequencing, they confirmed that children with different asthma phenotypes exhibited distinct dysbiotic patterns at different time points. Furthermore, they employed Phylogenetic Investigation of Communities by Reconstruction of Unobserved States to predict the functional potential of the fecal microbiota. This algorithm infers the functional metagenome of microbial communities from marker gene data and reference bacterial genomes, indicating that functional differences in the microbiota involve genes with diverse metabolic functions, and certain genes are organized into specific metabolic pathways. Specifically, asthma patients with the atopic + wheeze phenotype showed reduced abundances of *Lachnospira*, *Veillonella*, *Faecalibacterium*, and *Roseburia*. The functional differences in this community involve metabolic functional genes related to DNA replication, carbon metabolism, transporter proteins, and amino acid biosynthesis, with the lipopolysaccharide biosynthesis pathway being the most significant metabolic pathway [40].

### 8.2 Metagenomics: From Species Identification to Functional Potential Analysis

#### 8.2.1 Metabolomics

Metabolomics enables the high‐throughput detection of small molecule metabolites in feces, serum, or bronchoalveolar lavage fluid from asthma patients, identifying differential metabolites associated with asthma and gut microbiota dysbiosis. Furthermore, it allows for the precise quantification of key gut microbial metabolites, such as short‐chain fatty acids, secondary bile acids, and indole derivatives. By integrating metagenomic data, microbiome‐metabolome association analysis can be performed, directly linking the abundance of specific microbial genes with the concentrations of host endogenous and microbially derived metabolites, thereby substantiating causal relationships. A longitudinal study in Canada, by integrating SCFA levels in feces and urine with urinary metabolomic profiling, demonstrated significantly reduced acetate levels in asthma patients and identified differences in eight bacterially influenced urinary metabolites at 3 months of age in children who developed asthma, whereas only two were detected at 1 year, reflecting the impact of early‐life microbial dysbiosis. The study also confirmed higher excretion of sulfated bile acids glycolithocholate, glycocholenate, and glycohyocholate, along with reduced tauroursodeoxycholate, in asthmatic children. The most notable metabolic difference observed was a 14‐fold increase in urobilinogen. However, correlation analysis revealed no significant associations between microbial taxa and these metabolic changes, suggesting that these alterations in urinary metabolites may result from combined microbial and host metabolic activities. Nevertheless, they constitute markers of early‐life gut microbiota dysbiosis, detectable in urine, and are associated with asthma risk [40].

#### 8.2.2 Transcriptomics, Proteomics, and Immunomics

Transcriptomics enables RNA sequencing of host intestinal or pulmonary tissues, revealing alterations in the gene expression profiles of immune cells and epithelial cells under the influence of specific microbial communities. For instance, it can identify immune‐related gene networks modulated by specific microbial metabolites via G protein‐coupled receptors or histone deacetylases. Short‐chain fatty acids regulate epithelial barrier function and mucosal/systemic immunity through evolutionarily conserved processes involving G protein‐coupled receptor signaling or histone deacetylase activity. Studies indicate that asthma patients often exhibit reduced gut microbiota diversity, characterized by decreased abundance of *Bacteroidetes* and increased *Firmicutes* proportions, particularly associated with depletion of short‐chain fatty acid‐producing bacteria such as *Rothia* and *Faecalibacterium* [52]. Furthermore, proteomics and immunology utilize mass spectrometry to analyze protein profiles in serum or tissues, enabling quantification of cytokines, chemokines, immunoglobulins, and intestinal barrier proteins, thereby directly reflecting microbiota‐mediated immune and barrier functional status. Research by Esraah et al. demonstrates that the plant‐derived polyphenol resveratrol alleviates asthma via the gut‐lung axis by inducing beneficial microbiota and enhancing pulmonary barrier integrity; immunohistochemical analysis confirmed that resveratrol significantly increased tight junction proteins and reduced mucin in murine pulmonary epithelium, thereby reinforcing barrier structure [121].

## 9 Challenges and Future Directions

In recent years, the study of the two‐way regulatory mechanism of “gut‐lung” has revealed that the intestinal microbiota is involved in the immune regulation of asthma through multiple pathways, which provides a new breakthrough for the treatment of asthma, but there are still great challenges in the “gut‐lung” axis in bronchial asthma.

At the mechanistic level, although existing studies have delineated the role of gut microbiota dysbiosis and associated metabolites (such as short‐chain fatty acids, bile acids) in asthma pathogenesis, the specific signaling pathways of the gut‐lung axis (such as microbe‐neuro‐immune interactions) require further elucidation, and causal relationships between gut microbiota/gut‐lung axis and asthma remain under exploration. In addition, current research primarily focuses on the bacterial component of the microbiota, necessitating future systematic analyses of fungal communities, virome, and other microbial constituents to comprehensively unravel the regulatory mechanisms of the “gut‐lung” axis.

At the therapeutic level, the high interindividual heterogeneity of gut microbiota leads to inconsistent therapeutic outcomes, and specific molecular pathways underlying microbiota‐host interactions remain incompletely elucidated, necessitating the establishment of gut microbiota‐based asthma phenotypic stratification to guide personalized therapeutic strategies. Furthermore, existing therapeutic modalities such as fecal microbiota transplantation and helminthic therapy demonstrate limited efficacy in clinical translation for asthma, coupled with a paucity of targeted therapies specific to the “gut‐lung” axis. Future research should prioritize further elucidation of the mechanistic underpinnings of the “gut‐lung” axis and bridge fundamental experimental findings to clinical applications; research should control for baseline variations in individual microbiomes, including factors such as sex, age, ethnicity, comorbidities, medication use (such as antibiotics, probiotics), diet, lifestyle, and environmental exposures, to establish standardized measurement protocols for microbiome studies, validate findings in larger‐scale clinical datasets, and develop evidence‐based guidelines for microbiota‐targeted interventions.

## 10 Conclusion

The “gut‐lung” axis has become a research hotspot for asthma and other diseases. With the advancement of omics technology, a large number of clinical and animal studies on the treatment of asthma with intestinal microbiota have emerged, opening up a new model for the mechanism research and treatment of bronchial asthma, which can reshape the lung immune microenvironment by regulating the intestinal microbiota and its metabolites, and realize the transformation from “symptomatic treatment” to “etiological intervention”. Future studies should advance high‐quality clinical trials to validate the efficacy and safety of gut‐lung axis‐targeted therapies and promote personalized therapeutic approaches integrating the microbial‐immune‐metabolic triad.

## Author Contributions

**Ting Zheng:** conceptualization, writing – original draft, writing – review and editing, data curation. **Yi Huang:** writing – original draft. **Hongmei Yao:** writing – review and editing, supervision, resources, project administration, conceptualization.

## Funding

This work was supported by the Guizhou Provincial Basic Research Program Qiankehe Basic MS [2025] 506.

## Ethics Statement

This article is a review of previously published literature and does not contain any new studies with human participants or animals performed by any of the authors.

## Conflicts of Interest

The authors declare no conflicts of interest.

## Data Availability

Data sharing is not applicable to this article as no new data were created or analyzed in this study.
